# A biostimulant prepared from red seaweed *Kappaphycus alvarezii* induces flowering and improves the growth of *Pisum sativum* grown under optimum and nitrogen-limited conditions

**DOI:** 10.3389/fpls.2023.1265432

**Published:** 2024-01-08

**Authors:** Pushp Sheel Shukla, Nagarajan Nivetha, Sri Sailaja Nori, Sawan Kumar, Alan T. Critchley, Shrikumar Suryanarayan

**Affiliations:** ^1^ Research and Development Division, Sea6 Energy Private Limited, Centre for Cellular and Molecular Platforms, National Centre for Biological Sciences-Tata Institute of Fundamental Research, Bengaluru, Karnataka, India; ^2^ Verschuren Centre for Sustainability in Energy and the Environment, Sydney, NS, Canada

**Keywords:** *Kappaphycus alvarezii*, biostimulants, nitrogen metabolism, gene expression, *Pisum sativum*, sustainable agriculture

## Abstract

Nitrogen (N) is one of the critical elements required by plants and is therefore one of the important limiting factors for growth and yield. To increase agricultural productivity, farmers are using excessive N fertilizers to the soil, which poses a threat to the ecosystem, as most of the applied nitrogen fertilizer is not taken up by crops, and runoff to aquatic bodies and the environment causes eutrophication, pollution, and greenhouse gas emissions. In this study, we used LBS6, a *Kappaphycus alvarezii*-based biostimulant as a sustainable alternative to improve the growth of plants under different NO_3_^-^ fertigation. A root drench treatment of 1 ml/L LBS6 significantly improved the growth of *Pisum sativum* plants grown under optimum and deficient N conditions. No significant difference was observed in the growth of LBS6-treated plants grown with excessive N. The application of LBS6 induced flowering under optimum and deficient N conditions. The total nitrogen, nitrate and ammonia contents of tissues were found to be higher in treated plants grown under N deficient conditions. The LBS6 treatments had significantly higher chlorophyll content in those plants grown under N-deficient conditions. The root drench application of LBS6 also regulated photosynthetic efficiency by modulating electron and proton transport-related processes of leaves in the light-adapted state. The rate of linear electron flux, proton conductivity and steady-state proton flux across the thylakoid membrane were found to be higher in LBS6-treated plants. Additionally, LBS6 also reduced nitrogen starvation-induced, reactive oxygen species accumulation by reduction in lipid peroxidation in treated plants. Gene expression analysis showed differential regulation of expression of those genes involved in N uptake, transport, assimilation, and remobilization in LBS6-treated plants. Taken together, LBS6 improved growth of those treated plants under optimum and nitrogen-limited condition by positively modulating their biochemical, molecular, and physiological processes.

## 1 Introduction

Nitrogen is the most abundant mineral element required by plants growth and it is therefore one of the important limiting factors for their growth and yield (Weber and Burow, 2018; Bernard and Habash, 2009; Leptin et al., 2021). To increase agricultural productivity, farmers have intensively used nitrogen (N) mineral fertilizers applied directly to soils. The amount of synthetic nitrogen fertilizer usage has increased dramatically (McAllister et al., 2012). These applied synthetic N fertilizers pose risks to the ecosystem, as a very large portion is not used by crops and subsequently runs off to aquatic bodies, causing eutrophication (McAllister et al., 2012; Goñi et al., 2021). Additionally, these synthetics contribute significantly to N pollution and to greenhouse gas emissions (Liu et al., 2019). In addition, the raw materials for manufacturing these fertilizers are from finite sources, adding to the cost of agricultural produce (Manning, 2018). *Pisum sativum* is an economically important annual leguminous plant and known to rapidly acquire and assimilate nitrate during the reproductive stages due to tissue specific utilisation of nitrate and nitrogen (Singh and Dwivedi, 2023). Nitrogen starvation leads to the premature inhibition of flowering progression (Jeuffroy and Sebillotte, 1997). Therefore, despite the legume’s preference for nitrate uptake, pea significantly require N assimilation during the growth and reproductive stage (Jeuffroy and Sebillotte, 1997). Additionally, climate change and excessive use of the chemical fertiliser causes soil degradation leading to the loss of microbial diversity especially in the legumes adapted to the efficient use of nitrogen, and thus negatively impact the growth of plants (Vitousek et al., 1997; Savci, 2012; Jansson and Hofmockel, 2020).To compensate for these issues, researchers are focusing on the use of sustainable alternatives to improve the nitrogen-use-efficiency (NUE) in a variety crops, so there will be less requirement for the input of N fertilizer. Nitrogen is the major factor influencing flowering in plants (Lin and Tsay, 2017; Zhang et al., 2021). Higher and lower nitrogen contents delay flowering following a U-shaped curve, which means that tissue nitrogen content regulates flowering in a dose-dependent manner (Lin and Tsay, 2017). To reduce the application of chemical fertilizers and ensure sustainability in agriculture, an alternative approach of using naturally derived plant biostimulants is gaining ground in agricultural systems (White and Brown, 2010; Bodin et al., 2020; Shukla and Prithiviraj, 2021). These biostimulants are naturally derived from microbes, plants and seaweeds (Shukla et al., 2019).

Seaweeds are multitudinous (approximately 12,000 spp.) and many are naturally adapted to harsh habitats. These adaptation strategies involve the biosynthesis of unique biologically active compounds and secondary metabolites. Just a few of these compounds are currently commercially exploited to produce seaweed-derived, plant biostimulants as a novel class of sustainable agricultural inputs (Deolu-Ajayi et al., 2022). Most of the currently available candidates for the regulatory category of plant biostimulants are derived from a relatively small variety of seaweeds, in fact these are mostly from brown seaweeds, such as *Ascophyllum nodosum*, *Laminaria digitata*, *Ecklonia maxima*, and *Macrocystis pyrifera* (Shukla et al., 2016; Shukla et al., 2022). In general, seaweeds-based biostimulant extracts are rich sources of a wide range of natural and hydrolytically produced bioactive compounds (Shukla et al., 2016; Pereira et al., 2020). These biostimulants are reported to improve plant growth, as well as aspects of nutrient-use-efficiency, stress tolerance and defense (Shukla et al., 2018a; Shukla et al., 2018b; Jithesh et al., 2019; Sandepogu et al., 2019; Shukla et al., 2019; Shukla and Prithiviraj, 2021; Samuels et al., 2022; Shukla et al., 2023; Quille et al., 2022). Several reports have been published on the effects of brown seaweed extracts in improving nutrient uptake in plants (Shukla et al., 2018a; Shukla et al., 2019; Łangowski et al., 2022), e.g., Jannin et al. (2013) showed that AZAL5, a fucoid, *Ascophyllum nodosum*-derived biostimulant, improved nitrogen and sulfate uptake by regulating the expression of the genes involved in photosynthesis and nitrogen and sulfate metabolism. Similarly, the extract from *A. nodosum* improved nitrogen use efficiency in barley by upregulating the transporter involved in nitrate uptake in roots (Goñi et al., 2021). Another biostimulant from *A. nodosum* was shown to improve the nitrogen-use-efficiency of grass grown under nitrogen-limited conditions (Goñi et al., 2018).

A novel commercial biostimulant, i.e., LBS6, derived from the rhodophycean alga, *Kappaphycus alvarezii*, was engineered by Sea6 Energy Private Limited by the combination of different bioactive fractions present in juice extracted from pulping biomass and an acid hydrolysate of the pulp phase remaining after such a juicing extraction exercise (Nori et al., 2019; Shukla et al., 2023). The chemical composition of LBS6 was previously published by Shukla et al. (2023). The bioactive constituents of LBS6 comprise sulfated galacto-oligosaccharides within the defined molecular weight range of 400-10000 Da (Shukla et al., 2023) which have been demonstrated to improve plant growth and stress tolerance (Ravi et al., 2018; Arun et al., 2019; Banakar et al., 2022; Shukla et al., 2023). The application of LBS6 induced expansion of cotyledons by regulating the expression of genes involved in cell division, proliferation, expansion, and growth (Shukla et al., 2023). Foliar applications of LBS6 improved the growth of treated plants by regulating electron and proton transport-related processes (Shukla et al., 2023).

In contrast to our understanding of the response of biostimulants derived from brown seaweed in improving nitrogen-use-efficiency, little is known about the efficacy of *K. alvarezii*-derived biostimulants in inducing early flowering under nutrient-deprived conditions. In this study, we evaluated the efficacy of LBS6 in inducing flowering in *Pisum sativum* at a lower N fertigation rate than under normal conditions grown in an artificial soil and provide further insights to modes of action by studying the expressions of the genes specifically involved in N uptake and assimilation.

## 2 Materials and methods

### 2.1 Plant material and treatments

Seeds of common pea (i.e., *Pisum sativum*, variety Green fest) were purchased from Suttind Seeds Private Limited, Delhi, India. Four and a half-inch pots were filled with 550 g of Soilrite Mix TC (Keltech Energies Limited, Bengaluru, India), which is a mixture of Irish peat moss and horticultural grade perlite (75:25). In each pot, two seeds of *P. sativum* were sown at a depth of 2 cm. Seven days after sowing (DAT), the seedlings were thinned to one seedling per pot. The experiment was established to assess the effects of LBS6 on the growth of pea plants grown with ½ Hoagland, ½ Hoagland-Nitrogen, and ½ Hoagland-Nitrogen supplemented with 10 mM KNO_3_ as a nitrate source. Hoagland medium without nitrogen was procured from Himedia Laboratories, India (TS1117-5 L). The plants were drenched near the root zone with the 100 ml of treatment solutions on the 14^th^ and 21^st^ days after sowing. The details of treatments were listed in **Table 1**.

**Table 1:** List of different treatments.

Treatments	Description	
C1	Optimum nitrogen	½ Hoagland
C2		½ Hoagland+1 ml/L of LBS6
C3		½ Hoagland+0.5 ml/L of LBS6
T1	Nitrogen deficient	½ Hoagland-Nitrogen
T2		½ Hoagland-Nitrogen +1 ml/L of LBS6
T3		½ Hoagland-Nitrogen +0.5 ml/L of LBS6
T4	Excessive nitrogen	½ Hoagland-Nitrogen +10 mM KNO_3_
T5		½ Hoagland-Nitrogen +10 mM KNO_3 _+ 1 ml/L of LBS6
T6		½ Hoagland-Nitrogen +10 mM KNO_3 _+ 0.5 ml/L of LBS6

Hoagland solution was used as a source of macro and micronutrients (Nejamkin et al., 2020). The different treatments were arranged in a randomised block design on a metal bench in a greenhouse maintained at 24 ± 3°C by the fan and evaporative pad cooling system linked with temperature sensor and plants were grown under a natural day-light cycle. The plants were grown for 21 days after the second treatment (DAT). The average number of leaves per plant was counted 21 days after the second treatment (DAT). The rate of emergence of leaves was calculated by dividing the total number of leaves by the total number of days. Plant height was measured from the tip of the plant to the Soilrite using a graduated scale. For the fresh weight of shoots, the plants were uprooted and cut from the hypocotyl region and weighed on a digital analytical balance (Sartorius, Germany). The dry weight of the shoot was recorded by drying in a hot air oven at 72°C for 72 h. For the flowering, independent experiments were conducted by growing the plants under the different treatments until 45 DAT, and the plants showing the first flower bloom were recorded as the percentage of plants showing flowering. The total number of flowers was recorded at 45 DAT. Each experiment consisted of 10 replicates per treatment and was repeated in triplicate independently. The data were presented as an average value of 30 replicates per treatment.

### 2.2 Determination of chlorophyll a and b content

The effect of 0.5 and 1 ml/L of LBS6 on the chlorophyll a and b contents of leaves of *P. sativum* grown in Soilrite with optimum, deficient, and excessive nitrogen for 45 DAT was estimated according to the protocol described by Shukla and Prithiviraj (2021). Briefly, 500 mg of leaves from identical physiological positions were excised independently from 10 replicates from each treatment and frozen in liquid nitrogen. The leaves were ground in a mortar and pestle and suspended in 5 ml of cold 100% methanol in a 15 ml centrifuge tube. The suspension mixture was centrifuged at 10,000 rpm at 4°C for 10 min. The pellet was re-extracted twice with 5 ml of cold methanol until there was full discolouration of the pellet. The three fractions were combined, and the total volume was maintained at 15 ml with cold methanol. The absorbance of the extract was recorded at 652.4 and 665.2 nm using a UV-VIS spectrophotometer (NanoQuant, Tecan, Switzerland). The chlorophyll a and b contents were calculated as described by Lichtenthaler (1987). The values were presented as a mean of three replicates and was repeated thrice.


$$\text{Chl a}=16.72{\text{ Abs}}_{665.2}–9.16{\text{ Abs}}_{652.4}$$

$$\text{Chl b}=34.09{\text{ Abs}}_{652.4}–15.28{\text{ Abs}}_{665.2}$$

### 2.3 Determination of the nitrogen starvation index

Nitrogen deficiency induces chlorosis in plants, and this trait was used to calculate the nitrogen starvation index. A visual rating scale ranging from 1-5 was used for scoring chlorosis in pea leaves. The scoring scale was based on the visual estimation of chlorosis due to N deficiency in leaves as follows: 1, no chlorosis; 2, 1-25% leaf area showing chlorosis; 3, 25-50% leaf area showing chlorosis; 4, 50-75% leaf area showing chlorosis; and 5, 75-100% leaf area showing chlorosis. The nitrogen starvation index was calculated by using the following formula:


Nitrogen starvation index=Sum of the scoring/(Maximum possible score×Total number of leaves examined)

### 2.4 Determination of ammonia and nitrate content from the leaf samples

Ammonia in the leaf samples was estimated as a blue indophenol derivative under the catalytic influence of a nitroprusside salt (Bräutigam et al., 2007; Hachiya and Okamoto, 2017). Briefly, 500 mg of leaf tissues from the plants treated with different treatments (~500 mg) were ground in liquid nitrogen to a fine powder, extracted in 1 ml of 100 mM HCl, and added to 500 µl of chloroform. These were vortexed for 15 min at 4°C, and the aqueous phase was separated by centrifugation at 12000 × g for 10 min at 8°C. The aqueous phase was added to 50 mg of acid-washed activated charcoal, thoroughly mixed, and centrifuged (20000 g, 5 min, 8°C). The supernatant collected was used for the estimation of ammonia. For quantification, the supernatant was diluted 1:1 (v/v) in 100 mM HCl, and then 20 µl of this solution was mixed with 100 µl of solution containing 1% (w/v) phenol and 0.005% (w/v) sodium nitroprusside prepared in water and 100 µl of solution containing 1% (v/v) sodium hypochlorite and 0.5% (w/v) sodium hydroxide prepared in water. The reaction mixture was incubated at 37°C for 30 min, and absorbance was recorded at 620 nm using a UV-VIS spectrophotometer (NanoQuant, Tecan, Switzerland). The quantity of ammonia in the samples was estimated by comparison with the standard curve prepared using ammonium chloride and expressed as µmol/mg fresh weight. The experiment consisted of three replicates for each treatment and were performed in triplicates. The data was presented as an average of nine replicates.

Nitrate content in the leaf samples was measured as a yellow complex derivative under the nitration of salicylic acid by nitrate and subsequent addition of sodium hydroxide (Hachiya and Okamoto, 2017). Leaf samples (100 mg) were ground to a fine powder using liquid nitrogen and extracted with pre-heated, ultrapure water (80°C) by vortexing and incubating at 100°C for 20 min in a closed tube. The supernatant was collected by centrifugation (20400 g, 10 min, at room temperature). For quantification of nitrate, 100 µl of supernatant was added to 0.05% (w/v) salicylic acid in sulfuric acid, vortexed and incubated at room temperature for 20 min. Then, 1 ml of 8% NaOH was added to the reaction mixture, and absorbance was recorded at 410 nm using a UV-VIS spectrophotometer (NanoQuant, Tecan, Switzerland). The nitrate content was estimated by comparing a standard curve prepared with a known concentration of KNO_3_ and was expressed as µmol/g fresh weight. The experiment consists of three independent replicates for each treatment and were performed in triplicates. The data were presented as an average of nine replicates.

### 2.5 Determination of nitrogen, phosphorous and potassium content in leaves of *P. sativum*

The effect of 1 ml/L of LBS6 on the nitrogen, phosphorous and potassium contents in leaves of *P. sativum* grown with soilrite under optimum, deficient, and excessive nitrogen conditions for 42 days was determined as an outsourced service at ALS Testing Services India Private Limited, India, by following the protocol published in The Fertilizer (Control) Order, 1985 (https://www.faidelhi.org/).

### 2.6 Determination of total amino acid content in leaves of *P. sativum*

Total amino acid content in the leaves of the pea plants treated with 0.5 and 1 ml/L of LBS6 grown under optimum, deficient, and excessive nitrogen conditions were analysed by following the protocol described by Shukla et al. (2012). Briefly, 500 mg of the leaves was homogenized in 5 ml of 80% ethanol and centrifuged at 10000 rpm for 5 min. The pellet was re-extracted twice with 5 ml of 80% ethanol. All three fractions were combined, and the volume was maintained up to 15 ml. One hundred microliters of alcoholic extract were diluted to 1 ml and incubated with 1 ml of 0.2 M citrate buffer (pH 5), 1 ml of 80% ethanol, and 1 ml of ninhydrin (1%) at 95°C for 15 min. The samples were cooled, and absorbance was recorded at 570 nm using a UV-VIS spectrophotometer (NanoQuant, Tecan, Switzerland). The experiment consisted of three independent replicates for each treatment and were performed in triplicates.

### 2.7 Determination of malondialdehyde content in leaves of *P. sativum*

The malondialdehyde content in the leaves of the pea plants was estimated by following the methods described by Hodges et al. (1999). The alcoholic extract was prepared by homogenizing 500 mg of leaf tissue in 5 ml of cold 80% ethanol, followed by centrifugation at 5000 rpm at 4°C for 10 min. The pellet was re-extracted twice with 5 ml of 80% ethanol. All three fractions were combined, and the volume was maintained up to 15 ml. One hundred microliters of extracts from different treatments were diluted with 900 µl of water and mixed in a test tube in two sets: one with 1 ml of TBA solution containing 0.65% (w/v) TBA (thiobarbituric acid) with 20% (w/v) TCA and 0.01% (w/v) butylated hydroxytoluene (BHT) and another without TBA (thiobarbituric acid), including 20% (w/v) TCA and 0.01% (w/v) BHT. The resultant mixture was vortexed, incubated at 95°C in a dry bath for 25 min, cooled, and centrifuged at 5000 rpm for 10 min. The absorbance was recorded at 440, 532, and 600 nm. Malondialdehyde content was calculated using Equations 1–3:


(1) $$\left[\left({\text{Abs}}_{532+\text{TBA}}\right)–\left({\text{Abs}}_{600+\text{TBA}}\right)–\left({\text{Abs}}_{532-\text{TBA}}–{\text{Abs}}_{600-\text{TBA}}\right)\right]=\text{A},$$

(2) $$\left[\left({\text{Abs}}_{440+\text{TBA}}–{\text{Abs}}_{600+\text{TBA}}\right)0.0571\right]=\text{B},$$

(3) MDA equivalents (nmol/ml)=106[(A–B)/157,000]

### 2.8 Determination of electrolyte leakage in leaves of *P. sativum*

Nitrogen starvation-induced electrolyte leakage in the leaves of the LBS6-treated pea plants was estimated according to the protocol described in Yadav et al. (2012). The youngest fully expanded leaves from the same physiological position were collected from plants grown under different treatments for 42 days. The leaves were washed with distilled water and placed in a closed 50 ml falcon tube containing 10 ml of autoclaved distilled water. After 24 h of incubation at room temperature on a rotary shaker, the electrical conductivity (Lt) was measured using a conductivity meter (HI 3512 EC and resistivity meter (Hanna Instruments, USA)). The samples were autoclaved at 120°C for 20 min and cooled to room temperature to measure the electrical conductivity (Lo). The electrolyte leakage was measured according to Lutts et al. (1996).


Electrolyte leakage=(Lt/Lo)×100

### 2.9 Measurement of chlorophyll fluorescence and photosynthesis-related parameters

The photosynthesis-related parameters of treated plants was assessed by measuring chlorophyll fluorescence using MultispeQ V2.0 (PhotosynQ LLC, East Lansing, MI) associated with the PhotosynQ platform using the protocol Photosynthesis Rides 2.0 (www.photosynQ.org). The chlorophyll fluorescence measurement was recorded on the second and third fully expanded leaves of plants grown under optimum, deficient, and excessive nitrogen conditions, after 7 and 14 DAT of 0.5 and 1 ml/L of LBS6 and water as a control. Different photosynthesis-related parameters were recorded from two leaves per plant from the same physiological position. Each treatment consists of 10 independent replicates arranged in randomised block design and were performed in triplicate. Soil Plant Analyser Development (SPAD), linear electron flow (LEF), Fv’/Fm’ (efficiency of PSII in the light acclimatized state), Fs (steady state fluorescence), Phi2 (quantum yield of PSII electron transport), qL (fraction of PSII open centers), PhiNPQ (the fraction of light dedicated to nonphotochemical quenching), NPQ (nonphotochemical quenching) and PhiNO (the fraction of energy lost through nonregulated photosynthesis processes) were assessed in pea plants using MultispeQ V2.0.

The electron transport rate (ETR_PSII_), electrochromic band-shifts (ECSt), proton conductance of chloroplast ATP synthase (gH^+^), estimated proton flux through the thylakoid lumen (vH^+^), which represents the rate of ATP synthesis, and photosystem I (PS I) activity were estimated from the second and third leaves of plants grown under optimum, deficient and excessive nitrogen conditions, after 7 and 14 DAT of 0.5 and 1 ml/L of LBS6 and water as a control using MultispeQ V2.0 linked to the PhotosynQ platform using Photosynthesis Rides 2.0 (www.photosynQ.org). The fluorescence-based electron transport rate (ETR_PSII_) was estimated as described by Shukla et al. (2023). ETR_PSII_ was calculated using the quantum yield of PSII obtained by the pulse-amplitude modulation method at photosynthetically active radiation (PAR) of 500 μmol photons m^-2^s^-1^ as follows:


$${\text{ETR}}_{\text{PSII}}=0.84^{*}0.5^{*}\text{Phi}2^{*}\text{PAR}$$

### 2.10 Gene expression analysis by real-time PCR

The expression of the genes involved in N uptake, transport, assimilation, and remobilization in LBS6-treated plants grown under optimum, deficient, and excessive nitrogen conditions were analysed. The leaves were harvested 24, 48 and 72 h after the second treatment of 1 ml/L LBS6 and water (control), flash frozen in liquid nitrogen, and stored at -80°C. Total RNA was extracted from 100 mg of leaf sample by using TRIzol (Takara Bio, USA) and was quantified with a NanoDrop One (ThermoScientific, USA). cDNA was prepared from 1 µg of RNA with an iScript cDNA synthesis kit (Bio-Rad, USA). Real-time qPCR analysis was performed in Quant Studio5 (ThermoScientific, USA). The primer efficiency and specificity of amplification were evaluated by melt-curve analysis. The list of primers used in the study were presented **Supplementary Table S1**. Each experiment comprised three independent replicates and was repeated three times. The relative gene expression value was calculated using Livak’s method (2^-ΔΔCt^).

### 2.11 Statistical analysis

Plant phenotypic responses and gene expression data were evaluated with RStudio (Version 2023.06.1). The effect of different treatments was analysed by the one-way analysis of variance (ANOVA). When significant effects of treatments were found, multiple means comparison was carried out using Tukey’s HSD test, at p ≤ 0.05. The significantly different mean values are represented by different letters, obtained using the package ‘MultcompView’ (https://cran.r-project.org/package=multcompView). The data are expressed as average ± standard error (SE).

## 3 Results

### 3.1 Effect of LBS6 treatment on the growth of *P. sativum* plant under optimum, deficient and excessive nitrogen conditions

**Figure 1:**
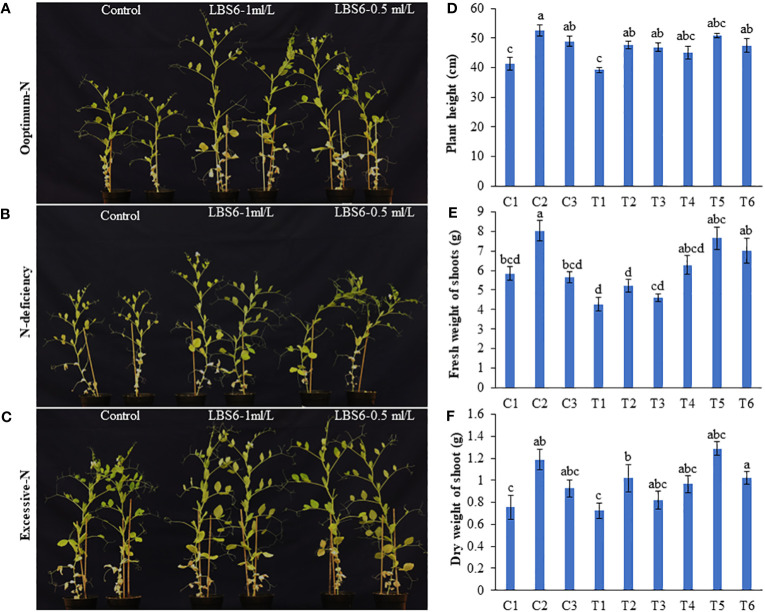
LBS6 improved the growth of *Pisum sativum* grown under different levels of nitrate supplementation. Pea plants were grown under **(A)** optimum-N, **(B)** N-deficient, and **(C)** excessive-N for 45 days. The plants were drenched near root-zone with water, LBS6 1mL/L and 0.5mL/L on 14th and 21st day after sowing. Effect of LBS6 on **(D)** Plant height, **(E)** fresh and **(F)** dry weight of shoots grown under N-limited conditions. Treatment details: plants are grown under optimum (C1, C2 and C3), N-deficient (T1, T2 and T3) and excessive-N (T4, T5 and T6) conditions. The plants sprayed with water (control) (C1, T1, T4), 1ml/L of LBS6 (C2, T2 and T5), and 0.5mL of LBS6 (C3, T3 and T6). The values were presented as mean ± SE and significantly different mean values at *p* ≤ 0.05 were represented by different letters. Each experiment was carried out in triplicate, and each experimental unit had ten plants (n=30).

**Figure 2:**
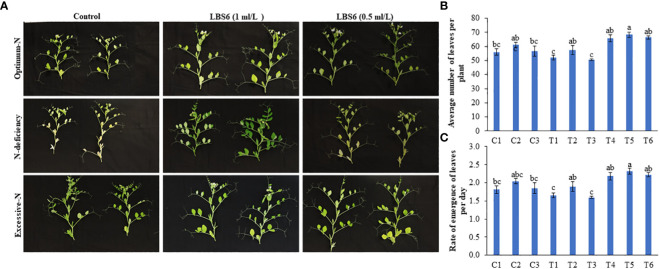
LBS6 root drenching positively influenced the rate of emergence of leaves of pea plants under under different nitrate supplementation. **(A)** The plastochron arrangement in 45 days old pea plants treated with water, LBS6 1mL/L and 0.5mL/L under different N contents. Effect of LBS6 on **(B)** Average number of leaves and **(C)** Rate of emergence of leaves. Plants are grown under optimum (C1, C2 and C3), N-deficient (T1, T2 and T3) and excessive-N (T4, T5 and T6) conditions. The plants sprayed with water (control) (C1, T1, T4), 1ml/L of LBS6 (C2, T2 and T5), and 0.5mL of LBS6 (C3, T3 and T6). The values were presented as mean ± SE and significantly different mean values at *p* ≤ 0.05 were represented by different letters. Each experiment was carried out in triplicate, and each experimental unit had ten plants (n=30).

The root drench of LBS6 improved the growth of the plants grown in Soilrite under optimum, deficient and excessive nitrogen conditions (**Figures 1A–F**, **2A, B**). The height of plant treated with 1 ml/L (C2) and 0.5 ml/L (C3) of LBS6 was found to be significantly increased by 27.23 and 18.45% compared to the control plants (C1). The plants grown under N-deficient condition (T1) showed a reduction in plant’s height compared to the plants grown with optimum nitrogen (C1) (**Figure 1D**). The plants treated with 1 and 0.5 ml/L LBS6 showed less of a detrimental effect of N deficiency on plant height. Supplementation with 1 and 0.5 ml/L LBS6 significantly increased plant height by 21.66 (T2) and 19.43% (T3), respectively, compared to untreated plants (T1) grown under N-deficient conditions (**Figure 1D**). The treatments of 1 and 0.5 ml/L LBS6 improved the height of the plants grown under and excessive N conditions, but the increment was not statistically significant. Similarly, the fresh weight of the plants irrigated with 1 ml/L LBS6 (C2) was significantly higher than that of the control plants (C1) under optimum-N content (**Figure 1E**). Under N-deficient conditions, the fresh weight was found to be higher in LBS6-treated plants at both concentrations, but the increase in the values was not statistically significant. Similarly, plants grown with excessive-N showed significantly higher fresh weight in 1 ml/L (T5) and 0.5 ml/L LBS6 (T6)-treated plants compared to the untreated plants (T4). The dry biomass of plants treated with 1 ml/L LBS6 was found to be significantly higher in the plants grown under different nitrogen contents (**Figure 1F**). The data presented in **Figure 1** clearly demonstrated that the root drench of the 1 ml/L of LBS6 improved the growth of the plants under optimum, and deficient- N conditions, though no significant difference was observed in the plants drenched with excessive N.

### 3.2 Effect of LBS6 on the rate of emergence of leaves of plants grown under optimum, deficient, and excessive nitrogen conditions

Under N deficient conditions, the plants treated with 1 ml/L LBS6 showed greener leaves and a higher number of leaves than the control plants (**Figures 2A, B**). The plastochron arrangement in LBS6-treated plants, under N-deficient conditions, was significantly different from that in untreated plants (**Figure 2A**). The average number of leaves in the 1 ml/L LBS6-treated plants grown under N-deficient conditions was significantly higher (10.4%) than that in the untreated plants grown under N-deficient conditions (**Figure 2B**). The average number of leaves was also found to be higher in LBS6-treated plants grown with optimum and excessive-N, but the changes in the values were not statistically significant (**Figure 2B**). The rate of emergence of the leaves per day was also found to be significantly higher in 1 ml/L LBS6-treated plants grown under N deficient conditions (**Figure 2C**). In all other treatments, no significant changes were observed in terms of the rate of emergence of leaves per plant.

### 3.3 Induction of early flowering in plants grown under optimum, deficient and excessive nitrogen conditions

**Figure 3:**
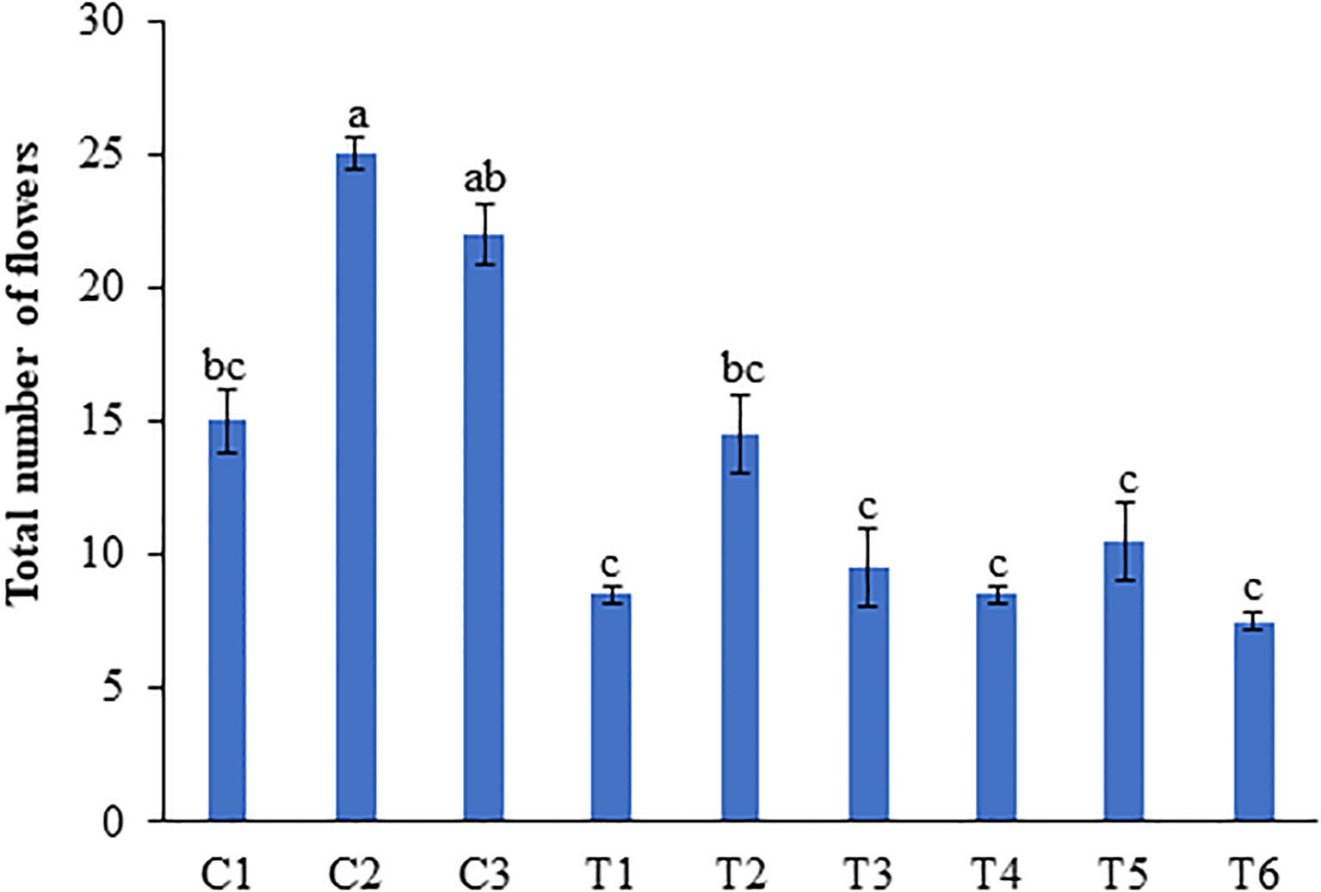
LBS6 application showed higher number of flowers in pea plants grown under different nitrate supplementation. Total number of flowers at 45 days after second treatment. Plants are grown under optimum (C1, C2 and C3), N-deficient (T1, T2 and T3) and excessive-N (T4, T5 and T6) conditions. The plants sprayed with water (control) (C1, T1, T4), 1ml/L of LBS6 (C2, T2 and T5), and 0.5mL of LBS6 (C3, T3 and T6). The values were presented as mean ± SE and significantly different mean values at *p* ≤ 0.05 were represented by different letters. Each experiment was carried out in triplicate, and each experimental unit had ten plants (n=30).

Treatment with 1 ml/L and 0.5 ml/L LBS6 induced earlier flowering in plants grown under N-deficient conditions. In the peas treated with 1 ml/L LBS6, 100% plant showed flowering at 39 DAT, while among the untreated plants, only 57.1% plants showed flowering at 39 DAT (**Supplementary Figure S1**). With optimal and excess N irrigation, no difference in the percentage of plants showing flowering was observed. After 45 days of the second treatment, the number of flowers per pot was counted (**Supplementary Figure S1**). The number of flowers was significantly higher in the 1 ml/L (C2) and 0.5 ml/L LBS6 (C3)-treated plants than in the control plants (C1) grown in Soilrite with optimum-N (**Figure 3**). The plants treated with 1 and 0.5 ml/L LBS6 showed 41.2 and 17.6% increases in the total number of flowers per plant respectively compared to control (**Figure 3**). Nitrogen starvation significantly reduced the total number of flowers per plant by 75%, but in LBS6-treated plants, the reduction in the total number of flowers was observed to be less than that in untreated plants (**Figure 3**). Under N-deficient conditions, the plants treated with 1 ml/L and 0.5 ml/L LBS6 showed 88% and 33.3% higher total numbers of flowers, respectively, compared to the untreated plants. Under excessive-N condition, 1 ml/L LBS6-treated plants showed a significantly higher (62.5%) number of flowers than the control plants, whilst no differences were observed between the 0.5 ml/L treated and control plants (**Figure 3**).

### 3.4 Effect of LBS6 on chlorophyll content in leaves of the plants grown under optimum, deficient and excessive nitrogen conditions.

**Figure 4:**
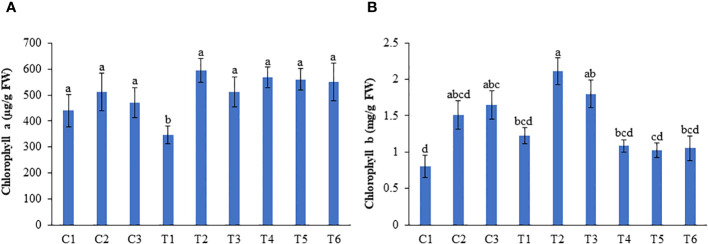
LBS6 improved the pigment content of pea plants grown under different nitrate supplementation. **(A)** Chlorophyll a and **(B)** Chlorophyll b content of pea leaves. Plants are grown under optimum (C1, C2 and C3), N-deficient (T1, T2 and T3) and excessive-N (T4, T5 and T6) conditions. The plants sprayed with water (control) (C1, T1, T4), 1ml/L of LBS6 (C2, T2 and T5), and 0.5mL of LBS6 (C3, T3 and T6). The values are presented as mean ± SE of three independent experiments which was repeated thrice (n=9), and significantly different mean values are represented by different letters.

No significant change in the chlorophyll a content was observed between LBS6-treated and untreated plants grown under optimum and excessive-N conditions. Under N-deficient conditions, 1 (T2) and 0.5 ml/L (T3) LBS6-treated plants showed higher chlorophyll a content than the control (T1). The plants treated with 1 and 0.5 ml/L LBS6 showed 71.2 and 47.25% higher chlorophyll a content than the untreated plants grown under N-deficient conditions (**Figure 4A**). The average chlorophyll b content in the leaves was found to be significantly increased by 88.6 and 5.5% in 1 and 0.5 ml/L LBS6-treated plants, respectively, compared with control plants grown under optimum-N condition (**Figure 4B**). Under N deficiency, 1 ml/L and 0.5 ml/L LBS6-treated plants showed higher (72.5% and 5.15%, respectively) average chlorophyll b content than the untreated plants. The plants grown under excessive-N showed no significant changes in the chlorophyll b content among the different treatments (**Figure 4B**).

### 3.5 Influence of LBS6 treatments on biochemical parameters in the leaves of plants grown under optimum, deficient and excessive nitrogen conditions

**Figure 5:**
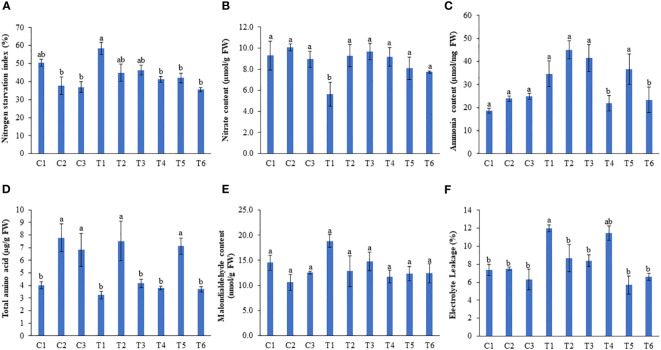
LBS6 regulates different biochemical parameters to improve plant growth under different nitrate supplementation. Effect of root drench with 0.5mL and 1mL/L of LBS6 on **(A)** Nitrogen starvation index, **(B)** Nitrate **(C)** Ammonia **(D)** Total amino acids and **(E)** Malondialdehyde content of leaves and **(F)** Electrolyte leakage. Plants are grown under optimum (C1, C2 and C3), N-deficient (T1, T2 and T3) and excessive-N (T4, T5 and T6) conditions. The plants sprayed with water (control) (C1, T1, T4), 1ml/L of LBS6 (C2, T2 and T5), and 0.5mL of LBS6 (C3, T3 and T6). The values are presented as mean ± SE of three independent experiments which was repeated thrice (n=9), and significantly different mean values are represented by different letters.

Nitrogen starvation index (NSI) was recorded by scaling the degree of chlorosis in plants. Nitrogen deficiency induced maximum chlorosis in peas, while those plants treated with 1 and 0.5 ml/L LBS6 showed reductions in chlorosis by 23.2 and 20.62%, respectively (**Figure 5A**). This is directly correlated with the significantly higher nitrate and ammonia contents in the leaves of the plants treated with 1 and 0.5 ml/L LBS6 grown under N-deficient conditions (**Figures 5B, C**). Additionally, the ammonia content was also found to be higher in LBS6-treated plants grown under N-deficient conditions, but the changes were not statistically significant (**Figure 5C**). No significant difference was observed in the nitrate and ammonia content in the leaves of the LBS6 treated and control plants grown under optimum and excessive-N conditions. A higher ammonia content represents higher amino acid biosynthesis in the leaves of the plants treated with LBS6 grown under N-starvation conditions (**Figure 5D**). In the plants under optimum-N condition, treatment of 1 (C2) and 0.5 ml/L (C3) of LBS6 showed significantly higher amino acid content in the leaves of treated plants as compared to untreated plants (C1). The 1 ml/L LBS6-treated plants grown under N-deficient conditions (T2) showed 28.14% higher total amino acid content compared to untreated plants (T1) (**Figure 5D**). The plants irrigated with excessive-N and supplemented with 1 ml/L LBS6 (T5) showed significantly higher total amino acid content compared to the plant grown only with excessive-N (T4) (**Figure 5D**). The MDA content was measured to determine N starvation-induced lipid peroxidation in the leaves of pea plants, which was significantly reduced in LBS6-treated plants (**Figure 5E**). N starvation-induced electrolyte leakage was also found to be significantly reduced in the LBS6-treated plants (**Figure 5F**). The application of excessive-N also induced electrolyte leakage in pea plants, while LBS6 supplementation resulted in a significant reduction in electrolyte leakage compared to untreated plants (**Figure 5F**).

### 3.6 Effect of treatment of LBS6 on photosynthesis-related parameters in leaves of plants grown under optimum, deficient and excessive nitrogen conditions

Leaf SPAD values are directly correlated with chlorophyll content in several crops. The plants treated with LBS6 (C2 and C3) showed significantly higher SPAD values than control plants (C1) under optimum-N condition (**Table 2**). N-deficiency resulted in a significant reduction in SPAD values (**Table 2**). LBS6-treated plants showed significantly higher SPAD values in the leaves of the plants grown under N starvation compared to the untreated plants at 14 DAT (**Table 2**). No significant differences were observed between the LBS6-treated and control plants under the excessive-N condition. Linear electron flow (LEF) was observed to be higher in the LBS6-treated plants under optimum-N at both time points, but the values were significantly higher in LBS6-treated plants after 14 DAT compared to the control (**Table 2**). Nitrogen deficiency conditions showed a significant reduction in LEF values in the untreated plants. The reduction in LEF value due to N deficiency was reportedly less in LBS6-treated plants at both time points (**Table 2**). After 7 DAT, no significant differences were observed in Fv’/Fm’ (the relative activity of PSII) and the quantum yield of PSII electron transport (Phi2) values in the plants grown under different treatments. However, after 14 DAT, LBS6-treated plants showed higher Fv’/Fm’ in the plants under optimum and N-deficiency compared to the respective control plants. Similarly, after 14 DAT, Phi2 values were higher in LBS6-treated plants grown in Soilrite under optimum and N-deficiency (**Table 2**). These data provide clear evidence that LBS6-treated plants improved the growth of plants under N deficiency by exciting the PSII biochemistry involved in photosynthesis. After 14 DAT, the loss of energy due to non-photochemical quenching was higher in N-deficient plants than in plants grown with optimum-N. LBS6-treated plants showed a significant reduction in the loss of energy in the form of nonphotochemical quenching in optimum and N-deficient conditions (**Table 2**). The fraction of light energy lost due to heat dissipation (ΦNPQ) and nonphotochemical quenching (NPQ) was significantly reduced in the plant plants grown under N-limited conditions. The LBS6 treatment did not show any difference in the values of the fraction of light energy lost due to the nonregulatory processes in plants grown under different treatments (**Table 2**).

**Table 2:** Effect of LBS06 on the chlorophyll fluorescence parameters from leaves of the pea plants grown under optimum, N-deficient and excessive-N conditions.

Parameters	Treatments	7 DAT	14 DAT
1. SPAD	C1	40.95 ± 1.08^ab^	31.40 ± 2.21^bc^
	C2	41.36 ± 0.85^ab^	38.41 ± 2.10^b^
	C3	42.79 ± 0.60^a^	36.28 ± 0.70^b^
	T1	35.77 ± 0.88^c^	23.91 ± 2.85^c^
	T2	37.64 ± 0.76^bc^	49.63 ± 1.52^a^
	T3	37.71 ± 1.30^bc^	31.78 ± 2.75^bc^
	T4	41.77 ± 0.94^ab^	40.83 ± 1.26^ab^
	T5	44.01 ± 0.79^a^	38.06 ± 0.57^b^
	T6	43.69 ± 0.58^a^	36.69 ± 2.63^b^
2. Linear Electron Flow	C1	32.36 ± 3.59^bc^	30.72 ± 5.60^abc^
	C2	49.31 ± 5.13^ab^	23.44 ± 5.09^c^
	C3	56.08 ± 4.32^a^	26.36 ± 5.64^bc^
	T1	28.18 ± 2.85^c^	22.11 ± 2.84^c^
	T2	41.07 ± 1.32^abc^	27.29 ± 3.74^bc^
	T3	36.67 ± 1.53^abc^	32.06 ± 2.47^abc^
	T4	46.25 ± 5.67^abc^	47.03 ± 4.77^ab^
	T5	39.21 ± 6.57^abc^	44.31 ± 5.06^abc^
	T6	39.93 ± 5.01^abc^	51.99 ± 6.71^a^
3. F_v_^’^/F_m_^’^	C1	0.69 ± 0.012	0.61 ± 0.025^bc^
	C2	0.68 ± 0.015	0.68 ± 0.015^ab^
	C3	0.69 ± 0.009	0.66 ± 0.011^ab^
	T1	0.68 ± 0.009	0.63 ± 0.014^bc^
	T2	0.67 ± 0.008	0.74 ± 0.004^a^
	T3	0.68 ± 0.007	0.67 ± 0.010^ab^
	T4	0.69 ± 0.014	0.62 ± 0.028^bc^
	T5	0.70 ± 0.013	0.54 ± 0.041^c^
	T6	0.70 ± 0.013	0.60 ± 0.021^bc^
4. Fs	C1	1323.39 ± 32.74^b^	1080.97 ± 91.59^c^
	C2	1322.42 ± 30.47^b^	1205.61 ± 31.50^bc^
	C3	1421.69 ± 16.20^ab^	1266.14 ± 40.13^abc^
	T1	1501.86 ± 43.76^ab^	1048.19 ± 67.50^c^
	T2	1413.89 ± 36.56^ab^	1524.53 ± 52.92^a^
	T3	1524.53 ± 52.92^a^	1413.89 ± 36.56^ab^
	T4	1390.00 ± 41.85^ab^	1221.42 ± 75.37^bc^
	T5	1396.53 ± 50.71^ab^	1022.31 ± 74.08^c^
	T6	1472.89 ± 61.98^ab^	1128.72 ± 76.16^bc^
5. Phi2	C1	0.54 ± 0.02	0.46 ± 0.05^bcd^
	C2	0.56 ± 0.02	0.59 ± 0.02^ab^
	C3	0.56 ± 0.02	0.54 ± 0.02^abcd^
	T1	0.56 ± 0.01	0.51 ± 0.02^abcd^
	T2	0.58 ± 0.01	0.61 ± 0.01^a^
	T3	0.57 ± 0.01	0.56 ± 0.01^abc^
	T4	0.56 ± 0.02	0.47 ± 0.03^bcd^
	T5	0.59 ± 0.02	0.42 ± 0.04^d^
	T6	0.56 ± 0.01	0.45 ± 0.03^cd^
6. ΦNPQ	C1	0.23 ± 0.02	0.3 ± 0.03^abcd^
	C2	0.22 ± 0.02	0.22 ± 0.02^cd^
	C3	0.23 ± 0.01	0.27 ± 0.02^bcd^
	T1	0.26 ± 0.01	0.31 ± 0.02^abc^
	T2	0.24 ± 0.01	0.16 ± 0.01^d^
	T3	0.24 ± 0.01	0.25 ± 0.01^bcd^
	T4	0.23 ± 0.02	0.34 ± 0.04^abc^
	T5	0.21 ± 0.02	0.38 ± 0.04^ab^
	T6	0.22 ± 0.01	0.43 ± 0.06^a^
7. Non-Photochemical Quenching	C1	1.13 ± 0.14	2.28 ± 0.41^abc^
	C2	1.03 ± 0.09	1.26 ± 0.17^bc^
	C3	1.11 ± 0.08	1.46 ± 0.13^bc^
	T1	1.28 ± 0.09	1.90 ± 0.19^bc^
	T2	1.32 ± 0.08	0.68 ± 0.04^c^
	T3	1.21 ± 0.07	1.36 ± 0.11^bc^
	T4	1.17 ± 0.17	2.12 ± 0.43^abc^
	T5	1.10 ± 0.15	2.35 ± 0.30^ab^
	T6	1.09 ± 0.16	3.77 ± 0.78^a^
8. PhiNO	C1	0.20 ± 0.005	0.17 ± 0.004^bc^
	C2	0.20 ± 0.006	0.17 ± 0.004^bc^
	C3	0.21 ± 0.004	0.19 ± 0.006^b^
	T1	0.19 ± 0.004	0.17 ± 0.005^bc^
	T2	0.20 ± 0.004	0.18 ± 0.005^b^
	T3	0.19 ± 0.004	0.23 ± 0.005^a^
	T4	0.20 ± 0.009	0.18 ± 0.012^b^
	T5	0.19 ± 0.006	0.14 ± 0.013^c^
	T6	0.21 ± 0.010	0.17 ± 0.005^bc^
9. Maximum amplitude of ECS signals (* 10^3^ mAU)	C1	0.62 ± 0.11	0.66 ± 0.11^abc^
	C2	0.77 ± 0.10	0.50 ± 0.09^c^
	C3	0.82 ± 0.09	0.61 ± 0.09^abc^
	T1	0.74 ± 0.07	0.59 ± 0.06^bc^
	T2	0.71 ± 0.05	0.57 ± 0.03^c^
	T3	0.62 ± 0.06	0.33 ± 0.04^c^
	T4	0.54 ± 0.08	0.61 ± 0.09^abc^
	T5	0.44 ± 0.07	1.04 ± 0.10^a^
	T6	0.59 ± 0.06	1.03 ± 0.15^ab^
10. Electron Transport Rate (ETR_PSII_)	C1	45.14 ± 7.62^abc^	28.67 ± 5.22^abc^
	C2	55.80 ± 4.88^a^	24.60 ± 5.26^bc^
	C3	51.46 ± 6.03^ab^	21.87 ± 4.75^c^
	T1	26.29 ± 2.66^c^	20.63 ± 2.64^c^
	T2	38.33 ± 1.23^abc^	29.92 ± 2.30^abc^
	T3	34.22 ± 1.43^abc^	25.47 ± 3.49^bc^
	T4	38.49 ± 5.30^abc^	43.89 ± 4.44^ab^
	T5	32.56 ± 6.31^bc^	48.52 ± 6.26^a^
	T6	51.41 ± 4.77^ab^	41.35 ± 4.71^abc^
11. Proton conductance of chloroplast ATP synthase (gH^+^)	C1	189.63 ± 28.70	127.24 ± 16.64
	C2	178.42 ± 27.75	115.94 ± 12.03
	C3	149.17 ± 14.17	107.80 ± 09.38
	T1	126.78 ± 09.71	085.74 ± 10.10
	T2	163.80 ± 19.18	122.10 ± 14.19
	T3	135.62 ± 09.26	129.67 ± 15.40
	T4	161.56 ± 16.17	142.47 ± 14.29
	T5	205.83 ± 25.02	103.09 ± 15.88
	T6	195.54 ± 15.24	123.47 ± 24.60
12. Proton flux through thylakoid membrane(vH^+^)	C1	0.09 ± 0.009	0.07 ± 0.010^abc^
	C2	0.11 ± 0.011	0.05 ± 0.014^abc^
	C3	0.10 ± 0.007	0.06 ± 0.009^abc^
	T1	0.08 ± 0.006	0.04 ± 0.007^bc^
	T2	0.10 ± 0.006	0.06 ± 0.007^abc^
	T3	0.09 ± 0.005	0.03 ± 0.003^c^
	T4	0.08 ± 0.012	0.07 ± 0.008^abc^
	T5	0.08 ± 0.008	0.09 ± 0.010^ab^
	T6	0.10 ± 0.009	0.09 ± 0.010^a^

Note: The letters following the values indicate the significant differences (Tukey’s HSD with 95% family-wise confidence level) at that specific time point.

The rate of linear electron transport through PSII in the light-adapted state (ETR_PSII_) was found to be significantly reduced in those plants grown in N-limited conditions compared to the plants grown with optimum nitrogen by 41.74% and 28.04% at both time points, while LBS6-treated plants showed a reduction in ETR_PSII_ by only 15% and 4% at both time points. ETR_PSII_ values in the LBS6-treated plants grown under N-deficient conditions were found to be significantly higher than those in the untreated plants (**Table 2**). The proton conductance of chloroplast ATP synthase (gH^+^) was also found to be reduced in plants grown under N-deficient conditions at both time points. After 14 DAT, LBS6 treatment showed significantly higher gH^+^ values in plants grown under N-starvation compared to untreated plants (**Table 2**). No statistically significant difference was observed in the proton flux through the thylakoid membrane (vH^+^) values of the LBS6 and water-treated plants grown under optimum, N-deficient and excessive-N conditions (**Table 2**).

### 3.7 NPK content in leaves of pea plants with LBS6 treatments grow under optimum, deficient, and excessive nitrogen conditions

Plants grown with 1 ml/L LBS6 showed better plant growth under different treatments; therefore, the N, P, and K contents were measured in the leaves of the pea plants treated with 1 ml/L LBS6 grown under optimum, N-deficient, and excessive-N conditions (**Supplementary Figure S2**). LBS6-treated plants under optimum-N (C2) showed significantly higher (59.5%) total N content than control plants (C1) (**Supplementary Figure S2A**). The total nitrogen content in the leaves of the plants was reduced by N starvation, but the LBS6-treated plants showed fewer symptoms of N deficiency, which was clearly visible by the 72.6% higher total N content in 1 ml/L LBS6 (T2)-irrigated plants than in the plants irrigated with water only under N-deficient condition (T1) (**Supplementary Figure S2A**). No significant difference in the total N content was observed under excessive-N condition, between the plants treated with 1 ml/L of LBS6 (T5) and control (T4) (**Supplementary Figure S2A**). Under N-deficient conditions, the P content in the leaves of the pea plants treated with 1 ml/L LBS6 was significantly higher than that of the untreated plants (**Supplementary Figure S2B**). The total K content was higher in the LBS6-treated plants than in control plants under both optimum and N-deficient conditions, but the increase was not statistically significant (**Supplementary Figure S2C**). Under excessive-N condition, both P and K content were reduced in LBS-6 treated plants compared to control (**Supplementary Figures S2B, C**).

### 3.8 Expression of genes involved in N uptake, transport, assimilation, and remobilization in the plants treated with LBS6 treatment under different nitrogen conditions

**Figure 6:**
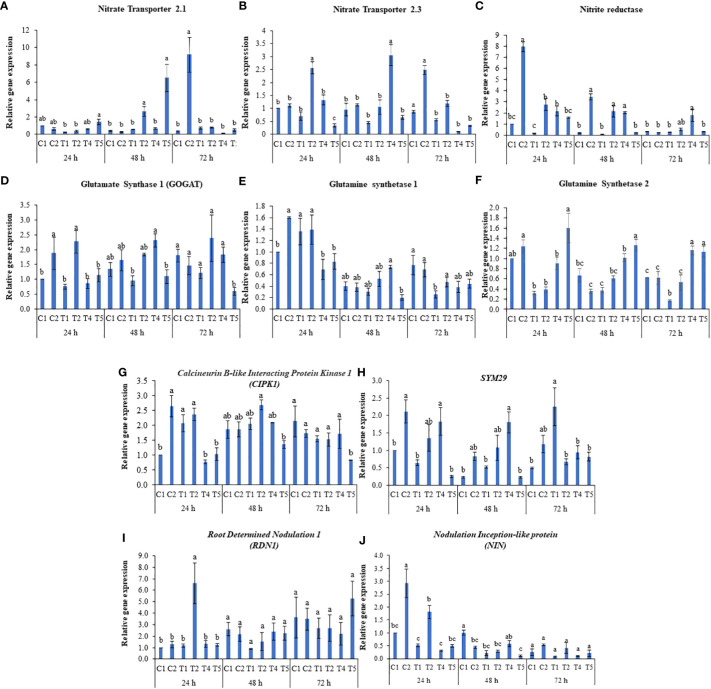
LBS6 regulate the expression of **(A)** *Nitrate Transporter 2.1 (NTR 2.1),* **(B)** *Nitrate Transporter 2.3 (NTR 2.3),* **(C)** *Nitrite Reductase (NiR)*, **(D)** Glutamate Synthase 1 (GOGAT), **(F)** *Glutamine Synthetase 1 (GS1)*, **(E)** *Glutamine Synthetase 2 (GS2)*, **(G)** *Calcineurin B-like Interacting Protein kinase 1 (CIPK1)*, **(H)** *SYM29*, **(I)** *Root Determined Nodulation 1 (RDN1)* and **(J)** *Nodulation Inception-like protein (NIN)* involved in N uptake, transport, assimilation, and remobilization under different nitrate supplementation. C1, T1 and T4 are control and C2, T2 and T5 are treated with 1ml/L of LBS6 under optimum, N-deficient, and excessive-N conditions respectively. The values are presented as mean ± SE of three independent experiments which was repeated thrice (n=9), and significantly different mean values are represented by different letters.

To further understand the role of LBS6 in improving plant growth under N-starvation conditions, the relative abundance of the transcripts of the genes involved in N-uptake, transport, assimilation, and remobilization was determined. The molecular study was conducted with control plants (C1, T1, T4) and plants treated with 1 ml/L LBS6 (C2, T2, T5) grown in Soilrite under optimum, deficient, and excessive-N conditions, respectively. After 72 h of LBS6 treatment, the expression of *NRT2.1* was 26-fold higher in treated plants than in control plants grown under the optimum-N condition (**Figure 6A**). Under N-deficient conditions, after 48 h of treatment, the transcripts of *NRT2.1* were found to be significantly higher in treated plants than in the control. Similarly, in plants grown under the excessive-N, after 48 h of LBS6 treatment, the expression of *NRT2*.1 was found to be 9.6 times higher than that in the untreated plants (**Figure 6A**). LBS6 significantly induced the expression of *NRT2*.3 in plants grown in optimum-N compared to control plants after 72 h of treatment (**Figure 6B**). The expression of *NRT2.3* was significantly induced 3.6 times after 24 h of treatment with LBS6 in the leaves of the plants grown under N-starvation compared to control plants (**Figure 6B**). Interestingly, after 24 and 48 h, significantly higher expression of NRT2.3 was observed in the plants grown under excessive-N compared to LBS6-treated plants grown under similar conditions, suggesting that LBS6 induces the expression of *NRT2*.3 under N-limited conditions but not in plant grown with excessive N (**Figure 6B**). The expression of *NiR*, which is involved in the conversion of nitrite to ammonia, was found to be 8.0- and 16.5-fold higher at 24 h and 48 h, respectively, in the LBS6-treated plants than in the control plants under optimum-N (**Figure 6C**). Under N-deficient conditions, LBS6-treated plants showed significant induction of *NiR* at 24 and 48 h by 13.6- and 20.5-fold, respectively, compared to control. Ammonia is assimilated in plants by glutamine synthetase (GS1) and glutamate synthase (GOGAT), which catalyze the conversion of α-ketoglutarate and ammonia to glutamate. Under optimum-N conditions, the expression of *GOGAT* was significantly induced after 24 h of treatment (**Figure 6D**). LBS6-treated plants showed significant induction of the expression of *GOGAT* gene in the plants grown under N-deficient conditions compared to untreated plants (**Figure 6D**), while in the presence of excessive-N, control plants showed higher expression of *GOGAT* compared to LBS6-treated plants (**Figure 6D**). The expression of *glutamine synthetase 1 (GS1)* and *glutamine synthetase 2* (*GS2*) was induced after 24 h of LBS6 treatment in plants compared to untreated plants under optimum-N (**Figures 6E, F**). Interestingly, at 24h of treatment, the expression of GS1 was found to be unaltered in the LBS6-treated and control plants grown under N deficient conditions as well as excessive N supplementation (**Figure 6E**), while *GS2* was significantly induced in the LBS6-treated plants compared to the untreated plants under excessive-N (**Figure 6F**). At 48h, the expression of *GS1* was significantly reduced in the LBS6-treated plants compared to the untreated plants under excessive-N, while no significant changes was observed in other treatments. The expression of *GS1* was significantly induced after 72h of treatment in LBS6-treated plants as compared to untreated plants grown under N deficient conditions. Calcineurin B-like interacting protein kinase was associated with the expression of NRT and was reported to be modulated by LBS6 treatment (**Figure 6G**). There was significant induction of *CIPK1* after 24 h of LBS6 treatment in those plants, as compared to the control plants under optimum-N. Under N-deficient conditions, the expression of CIPK1 was higher in the LBS6-treated plants at 24 and 48 h, but the increase was not statistically significant (**Figure 6G**). In the plants grown with excessive N, the expression of CIPK1 was found to be higher in the control plants than in the LBS6-treated plants at 48 and 72h treatment (**Figure 6G**). *SYM29*, an orthologue of *hypernodulation aberrant root* (*har1*) involved in nitrate-dependent shoot nodulation in legumes, was found to be significantly induced in the LBS6-treated plants grown with an optimum dose of N. Under N-deficient conditions, initially 24 and 48 h, the expression of *SYM29* was higher in LBS6-treated plants, but after 72 h of LBS6 treatment, the expression of SYM29 was significantly reduced by 3.35 times in treated plants compared to untreated plants (**Figure 6H**). The expression of the *RDN1 (root dertermined nodulation 1)* gene, which is involved in the long-distance signaling system for higher nodulation, was significantly induced after 24 h of LBS6 treatment in the plants grown under N starvation (**Figure 6I**). In the plants grown under excessive-N, the expression of *RDN1* was found to be significantly higher in LBS6-treated plants at 72 h than in untreated plants (**Figure 6I**). The transcripts of *PsNIN* were significantly increased after 24 h of LBS6 treatment in the plants compared to the untreated plants, under optimum and N-deficient conditions, while at all other time points, no significant difference was observed (**Figure 6J**). These results clearly demonstrated that LBS6 differentially modulates the expression the expression of genes involved in NO_3_^-^ uptake and assimilation in plants grown under optimum, deficient and excessive N conditions.

## 4 Discussion

The root drench application of LBS6, at a dose rate of 1 ml/L, improved the growth of pea plants even with the optimal level of NO_3_^-^ present in ½ Hoagland (approximately 5 mM). Under N-deficient conditions, the LBS6-treated plants showed better growth in terms of plant height, rate of emergence of leaves, leaf number and greenness of the leaves. Flowering induction in plants is largely dependent on optimum nitrogen levels (Lin and Tsay, 2017; Zhang et al., 2021). High supplementation of N, as well as its limitation, delays flowering in plants (Zhang et al., 2021). Supplementation with LBS6 resulted in early induction of flowering in plants grown under N-deficient conditions. LBS6-treated plants exhibited a significantly higher number of flowers per plant grown under optimum and N-deficient conditions. Nitrogen is the most mobile element, and its deficiency induces chlorosis in older leaves compared with younger leaves. Nitrogen deficiency induces chlorosis in the plants, while the LBS6-treated plants showed less N- starvation induced chlorosis, which was clearly represented by higher chlorophyll content in LBS6-treated plants grown under the optimum, N-deficient, and excessive-N conditions. N starvation-induced chlorosis led to the higher generation of reactive oxygen species (ROS) (Dong et al., 2023). LBS6-treated plants grown under optimum and N-deficient conditions showed less malondialdehyde content, produced as a biproduct of lipid peroxidation of the membrane due to ROS formation (Dong et al., 2023). LBS6-treated plants showed reduced electrolyte leakage under N-deficient, suggesting reduced N starvation-induced ROS accumulation.

Chlorophyll in leaves is directly proportional to the amount of light energy trapped to fix atmospheric carbon dioxide (Kandel, 2020). N starvation in leaves inhibits chlorophyll biosynthesis and consequently reduces photosynthetic efficiency (Antal et al., 2010). SPAD values represent the chlorophyll content and were significantly higher in the LBS6-treated plants grown under N-deficient and hence more absorption capacity of light energy. N starvation limits the quantum yield of PSII and impacts the plant’s photochemistry (Tantray et al., 2020). Supplementation of those peas with LBS6 resulted in higher PSII activity under N-deficient in terms of the efficiency of PSII in the light-acclimatized state (Fv’/Fm’) and the quantum yield of PSII electron transport (Phi2). Additionally, LBS6 supplementation prevented the loss of photosynthetic radiation in the form of heat energy (ΦNPQ) and non-photochemical quenching due to N starvation compared to control. The electron flow via the various electron receptors in the thylakoid membrane is generated by the photosynthetically active radiation (PAR) absorbed by reaction centers of PSII and is utilized to produce ATP and NADPH, which are employed in the Calvin cycle for CO_2_ fixation (Yamori et al., 2015; Shukla et al., 2023). N starvation reduces ETR_PSII,_ leading to the reduced ATP and NADP production required for CO_2_ fixation during the Calvin cycle. Supplementation with LBS6 resulted in higher ETR_PSII_ in those plants grown under N-deficient conditions, thus improving the capacity of treated plants to produce more ATP and NADPH. The dark interval relaxation kinetics (DIRK) of the electrochromic band shift in the plants grown with optimum, deficient and excessive nitrate were measured by MultispeQ and used to calculate the proton motive force (ECSt), proton conductance of chloroplast ATP synthase (gH^+^), and estimated proton flux through the thylakoid lumen (vH^+^), which represents the rate of ATP synthesis (Avenson et al., 2004; Tantray et al., 2020; Ibrahimova et al., 2021). The root application of LBS6 showed higher values of gH^+^ and vH^+^, resulting in the higher generation of ATP in treated plants under N starvation and hence better plant growth. These results provide clear evidence that supplementation with LBS6 protect the plants from the deleterious effects of N starvation by improving PSII-related photochemistry.

In this work, a significant increase in nitrate and ammonia content was found in the LBS6-treated plants grown under optimum, and deficient N conditions. Nitrates are the main source of N for the plants (Orsel et al., 2002; Stitt, 1999), and NRT1 and NRT2 are the major classes of high- and low-affinity transporters, respectively (Orsel et al., 2002; Parker and Newstead, 2014). The root drench treatment of LBS6 significantly induced the expression of *NRT2*.1 and *NRT2*.3 in the pea plants grown with optimum and deficient N conditions, suggesting an increased NO_3_^-^ absorption capacity of treated plants. The higher nitrate accumulation in the LBS6-treated shoots provides evidence that LBS6 improves the nitrate transport ability by regulating the expression of CIPK1, which phosphorylates nodule inception (NIN)-like protein and translocation to the nucleus to induce the transcription of assimilatory proteins such as nitrite reductase (NiR) and the transporter PsNRT2.3 (Goñi et al., 2021; Gu et al., 2022). Higher nitrate in LBS6-treated plants under N-deficient conditions induces the expression of the regulatory gene *PsNIN*, which regulates the expression of genes involved in nitrate uptake and assimilation (Castaings et al., 2009; Gu et al., 2022). In pea, SYM29, a putative serine/threonine receptor kinase, regulates root growth and is required for nitrate sensitivity for symbiotic interactions (Krusell et al., 2002). In this study, LBS6-treated plants showed higher expression of *SYM29*, explaining the plausible reason behind the better growth in LBS6-treated plants under optimum N and N-deficient conditions. Additionally, the nitrate level in the root regulates auxin transport activity by differentially regulating the expression of NRT2.1 and thus plant growth (Wang et al., 2023). Nitrate is reduced to nitrite in the cytosol by nitrate reductase, which is then transported to the chloroplast and converted to ammonia by nitrite reductase (NiR) (Orsel et al., 2002). The root drench application of LBS6 showed induction of *NiR* transcripts in the plants grown under optimum N and N-deficient conditions, suggesting higher accumulation of ammonia, which is converted to amino acids by the GS/GOGAT cycle (Temple et al., 1998; Gu et al., 2022). Higher amounts of ammonia are converted to α-ketoglutarate by glutamine synthetase (GS1 and GS2) and glutamate synthase (GOGAT) in plants, which are used in different biochemical reactions and amino acid biosynthesis (Temple et al., 1998; Bernard and Habash, 2009). LBS6 differentially modulates the expression of GS1, GS2 and GOGAT to improve the growth of plants under optimum-N, N-deficient and excessive N conditions. The total amino acid content was significantly higher in the plants treated with LBS6 grown under optimum-N, N-deficient and excessive N conditions. These results clearly showed that the bioactive molecules present in LBS6 improved the growth of pea plants under optimum and N-limited conditions by regulating the expression of the genes involved in nitrate uptake and assimilation and modulated various biochemical parameters to mitigate deleterious effects of N deficiency.

## 5 Conclusion

**Figure 7:**
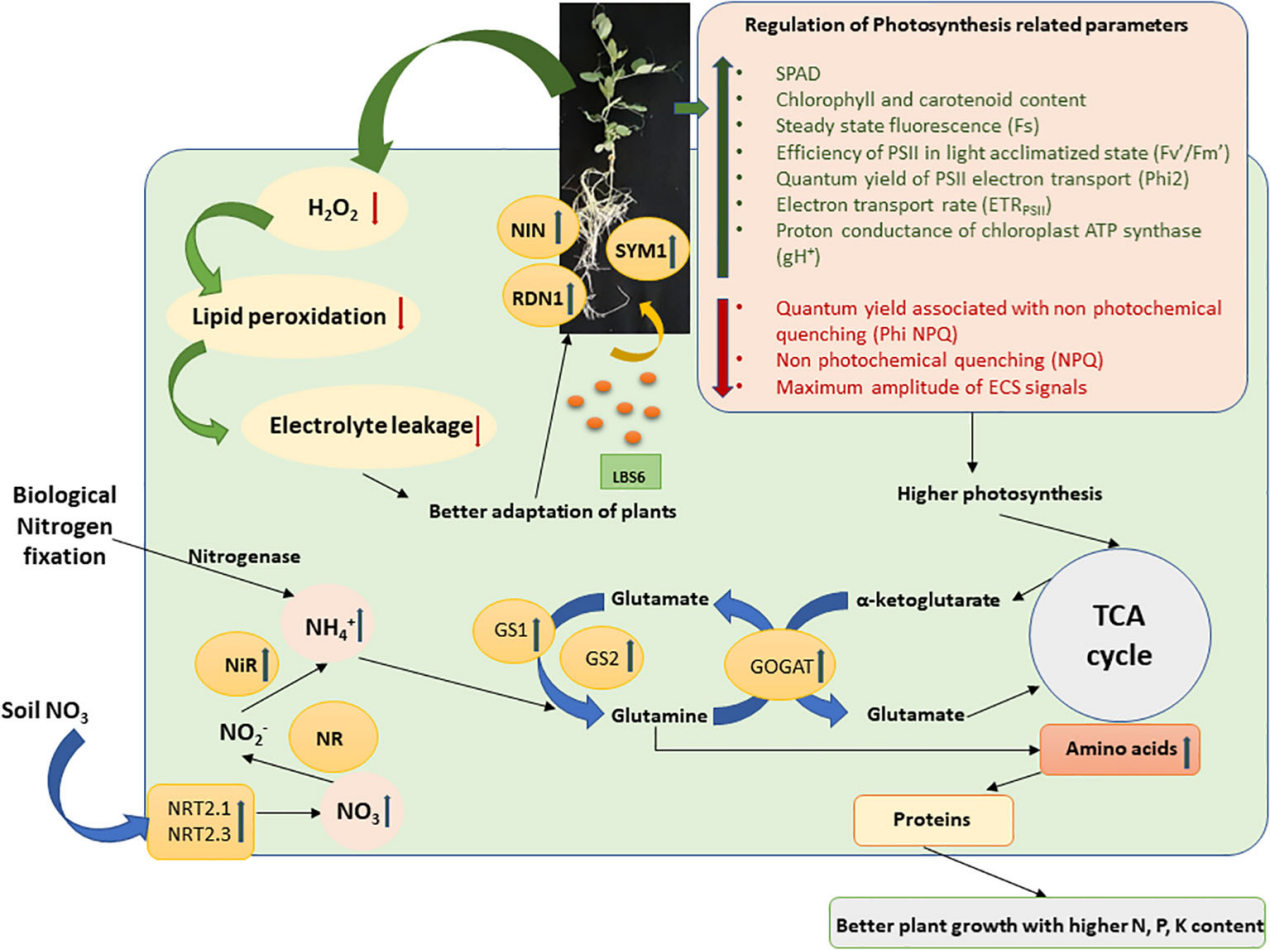
A schematic diagram of regulation of N-uptake and assimilation by the root drench application of LBS6 under N limited conditions. The green arrow shows the upregulation of the gene expression and enzyme activity, whereas the red arrow shows the downregulation.

The bioactive compounds present in LBS6, a red seaweed-derived biostimulant improved the inherent capacity of the plants to absorb more nitrate and assimilate it to usable organic forms in the plants. This study investigated the hypothesis of reducing the dependency on synthetic chemical fertilizers by judicious introduction of red seaweed-based biostimulants in nutrient management programs. This study provided an understanding of the mode of action of LBS6 in improving growth and inducing flowering in plants grown under optimum and N deficient conditions. The results presented in this study showed that the maximum biostimulatory activity of LBS6 was observed when the plants were grown in optimum N and N-deficient conditions, while little differences was observed in case of excessive N. **Figure 7** represents a holistic mechanism by which LBS6 improved the growth of peas by regulating different physiological, biochemical, and molecular processes. The results presented in this study provide a basis for the practice of using LBS6 as a sustainable strategy for improving nutrient uptake in plants. However, to include LBS6 in farmers’ practice of nutrient management programs, additional studies must be conducted on different crop systems under field conditions which are reported to be deficient in mineral nutrients.

## Data availability statement

The original contributions presented in the study are included in the article/**Supplementary Material**. Further inquiries can be directed to the corresponding authors.

## Author contributions

PS: Conceptualization, Data curation, Formal analysis, Investigation, Methodology, Project administration, Supervision, Validation, Visualization, Writing – original draft, Writing – review & editing. NN: Data curation, Formal analysis, Methodology, Validation, Writing – review & editing. SN: Conceptualization, Funding acquisition, Project administration, Supervision, Writing – review & editing. SK: Conceptualization, Supervision, Writing – review & editing. AC: Conceptualization, Supervision, Writing – review & editing. SS: Conceptualization, Funding acquisition, Project administration, Writing – review & editing.
