# Supplementary material for: A biostimulant prepared from red seaweed Kappaphycus alvarezii induces flowering and improves the growth of Pisum sativum grown under optimum and nitrogen-limited conditions
Source: Front Plant Sci. 2024 Jan 8;14:1265432. doi: 10.3389/fpls.2023.1265432 (PMC10951999; doi:10.3389/fpls.2023.1265432)
Supplement: Supplementary file 1 [file DataSheet_1.pdf]

Supplementary Material

AgroGain®, a biostimulant prepared from *Kappaphycus alvarezii* induces flowering and improve growth of *Pisum sativum* grown under optimum and nitrogen limited conditions.

Pushp Sheel Shukla[1*], Nagarajan Nivetha [1], Sri Sailaja Nori[1*], Sawan Kumar, Alan Critchley[2], Shrikumar Suryanarayan[1]

[1]Research and Development Division, Sea6 Energy Private Limited, Centre for Cellular and Molecular Platforms, National Centre for Biological Sciences-Tata Institute of Fundamental Research, Bengaluru, Karnataka, India

***Correspondence:**
Pushp Sheel Shukla, pushp.shukla@sea6energy.com

Sri Sailaja Nori, sailaja@sea6energy.com

1.1 Supplementary Figure

Supplementary Figure S1. LBS6 induces early flowering in pea plants grown under different Nitrate supplementation Pea plants were grown till 45 days after second treatment (DAT) with water LBS6 1mL/L and 0.5mL/L, and the plants showing the first flower blossom were recorded as percentage of plants showing flowering. The colors ranging from red to green represent the average values of percentage of plants showing flowering (red, low percentage and green, high percentage). The values were presented as a mean of three replicate and each replicate consists of 10 plants.

Percentage of plants showing flowering

Number of days	C1	C2	C3	T1	T2	T3	T4	T5	T6
31 DAT	0.0	0.0	0.0	0.0	0.0	0.0	0.0	0.0	0.0
32 DAT	0.0	0.0	0.0	0.0	14.3	14.3	0.0	0.0	0.0
33 DAT	0.0	0.0	0.0	0.0	14.3	14.3	0.0	0.0	0.0
34 DAT	0.0	0.0	0.0	0.0	28.6	57.1	0.0	0.0	0.0
35 DAT	0.0	0.0	0.0	0.0	42.9	71.4	0.0	0.0	0.0
36 DAT	0.0	14.3	0.0	0.0	71.4	85.7	0.0	0.0	0.0
37 DAT	0.0	14.3	0.0	28.6	71.4	85.7	0.0	0.0	0.0
38 DAT	14.3	28.6	0.0	28.6	85.7	100.0	0.0	0.0	0.0
39 DAT	14.3	28.6	0.0	57.1	100.0	100.0	14.3	0.0	0.0
40 DAT	14.3	28.6	0.0	71.4	100.0	100.0	14.3	0.0	0.0
41 DAT	42.9	42.9	0.0	71.4	100.0	100.0	28.6	0.0	0.0
42 DAT	42.9	57.1	28.6	85.7	100.0	100.0	28.6	0.0	14.3
43 DAT	57.1	57.1	42.9	100.0	100.0	100.0	28.6	28.6	28.6
44 DAT	57.1	57.1	57.1	100.0	100.0	100.0	42.9	28.6	28.6
45 DAT	57.1	57.1	57.1	100.0	100.0	100.0	42.9	42.9	28.6

Supplementary Figure S2. Mineral content in the leaves of *Pisum sativum* grown under N-starvation and excessive conditions. C1, T1 and T4 are control and C2, T2 and T5 are treated with 1ml/L of LBS6 under optimum, N-deficient, and excessive-N conditions respectively. The values in graph represent the relative gene expression. The values are presented as mean ± SE of three independent replicates, and significantly different mean values are represented by different letters.

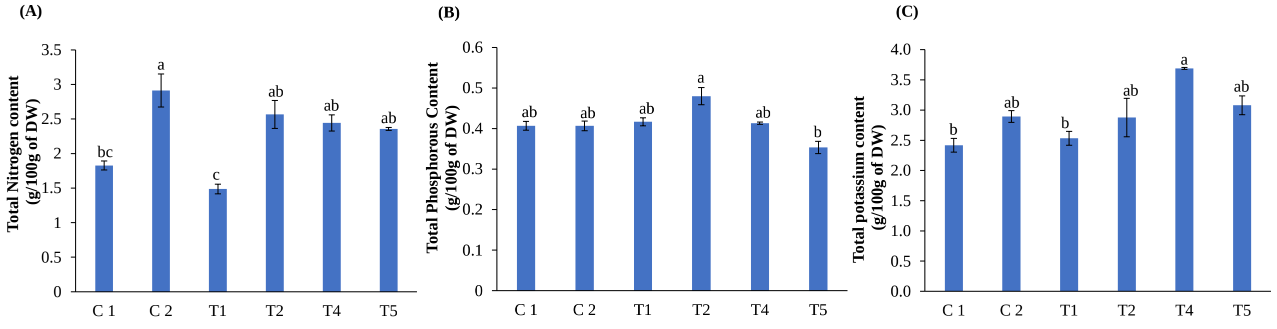

Supplementary Table S1. List of primers used in this study.

Gene name		Primer sequence
Nitrate Transporter 2.1	*PsNTR2.1 F*	TTGCGCTGCTTTACGGTTAC
	PsNTR2.1 R	TGGCCATTCCAAAGATGCAG
Nitrate Transporter 2.3	*PsNRT2.3 F*	TTGGAGTGGAGTTGACTGTGG
	PsNRT2.3 R	TGCAGCAATGATTCCAGCAG
Nitrite reductase	*PsNIR F*	TTCTCACCAGAACCATCCATCC
	PsNIR R	TTATCGCTTGGCCGCAAAAC
Glutamate Synthase 1 (GOGAT)	*PsGLT1F*	AGAGGGAAGACAAGCTGCTTC
	PsGLT1R	TGCCCTTGTGCTTGTTGTTG
Glutamine synthetase 1	*PsGlU1 F*	AGCTGGAATTGGCACTGTTG
	PsGlU1 R	ACCTCCTGCATGTTTGATGG
Glutamine Synthetase 2	*PsGS2F*	TGAGACAGCCAGCATTAACG
	PsGS2R	TTGCCATTCTTCTCGGTGTC
Calcineurin B-like interacting protein kinase	*PsCIPKF*	AGGACCTCTCGGTTTTGATGTC
	PsCIPKR	ACCTTTCCGTCCAGTCTTTTCC
Symbiosis 29 (An orthologue of hyper-nodulation aberrant root (har1))	*PsSYM1 F*	GCTTGACGCAAATCAGTTCG
	PsSYM1 R	TGAAGTCAACGGCTGTCAAC
Root determined nodulation 1	*PsRDN1 F*	GGCAATTCCCCCGTTATAATCC
	PsRDN1 R	TCAAGCACCCATCCGAAAAC
Nodulation Inception (NINs)	*PsNIN F*	AACAAAGGGAAAGGCGTGTC
	PsNIN R	TCTCCCAGTTACAGGTTGTGAC
